# Human papillomavirus vaccination hesitancy among young girls in Ethiopia: factors and barriers to uptake

**DOI:** 10.3389/fpubh.2025.1507832

**Published:** 2025-01-23

**Authors:** Ashenafi Kibret Sendekie, Biruk Beletew Abate, Betelhem Anteneh Adamu, Aschalew Mulatu Tefera, Kaleab Temelket Mekonnen, Melkam Alemu Ashagrie, Yabibal Berie Tadesse, Abera Dessie Dagnaw, Mequannent Sharew Melaku, Gizachew Kassahun Bizuneh

**Affiliations:** ^1^Department of Clinical Pharmacy, School of Pharmacy, College of Medicine and Health Sciences, University of Gondar, Gondar, Ethiopia; ^2^School of Pharmacy, Curtin Medical School, Faculty of Health Sciences, Curtin University, Bentley, WA, Australia; ^3^College of Medicine and Health Sciences, Woldia University, Woldia, Ethiopia; ^4^School of Population Health, Curtin University, Bentley, WA, Australia; ^5^Departement of Pharmacognosy, School of Pharmacy, College of Medicine and Health Sciences, University of Gondar, Gondar, Ethiopia; ^6^Department of Pharmaceutical Chemistry, School of Pharmacy, College of Medicine and Health Sciences, University of Gondar, Gondar, Ethiopia; ^7^Department of Health Informatics, Institute of Public Health, College of Medicine and Health Sciences, University of Gondar, Gondar, Ethiopia

**Keywords:** Human papillomavirus, HPV vaccine, vaccine hesitancy, vaccination acceptance, knowledge, attitude, young girls, Ethiopia

## Abstract

**Background:**

Human papillomavirus (HPV) vaccinations protect against HPV infections. The infection might lead to vaginal cancer, vulvar cancer, genital warts, cervical intraepithelial neoplasia lesions, and cervical cancer. This study assessed hesitancy of HPV vaccination, associated factors, and barriers to vaccination among youth girls in Ethiopia.

**Methods:**

An institutional-based cross-sectional study was conducted among female undergraduate students at the University of Gondar, College of Medicine and Health Sciences, between July and August 2022. The data was collected using a self-administered questionnaire. A simple random sampling method was used to recruit participants. The data were entered and analyzed with SPSS version 26. Descriptive statistics were used to describe the participants’ demographic characteristics. Logistic regression was performed to identify the significant factors associated with acceptance of the HPV vaccine. A *p*-value <0.05 was considered statistically significant.

**Results:**

The study included 423 participants with a mean age of 22.5 ± 6.7 years. Only more than one-third (35.2, 95% CI: 27.2–44.1) received the HPV vaccine. Currently, more than one-fourth (27.9, 95% Cl: 21.4–33.8) of participants are hesitant to receive the HPV vaccine. Higher monthly income (AOR = 1.52, 95% CI: 1.08–6.34), good knowledge of the HPV vaccine (AOR = 2.12, 95% CI: 1.12–4.87), and a positive attitude towards the vaccine (AOR = 3.03, 95% CI: 1.63–9.56) were significantly associated with acceptance of HPV vaccination. Safety concerns (63.1%), misinformation (42.8%), and parental concerns (42.3%) about the HPV vaccine were among the top perceived reported barriers to receiving the HPV vaccine.

**Conclusion:**

This result showed that more than a quarter number of youth girls are still hesitant to receive HPV vaccinations. To increase vaccination acceptance, interventions should focus on awareness-raising programs about HPV infection and vaccines and addressing safety and parental concerns.

## Introduction

Human papillomavirus (HPV) is a common sexually transmitted infection (STI) that can cause a variety of health problems, including cervical cancer, genital warts, and oropharyngeal cancer (1). The World Health Organization (WHO) estimates that approximately 630,000 women worldwide develop cervical cancer each year, resulting in 317,000 deaths. Most of these deaths occur in low- and middle-income countries, including Ethiopia. More than 85% of cases are diagnosed in developing countries (2). In Ethiopia, cervical cancer is the second most common cause of cancer death in women, with an age-specific incidence and mortality rate of 21.5 and 16 deaths per 100,000 females, respectively (3, 4).

The development of HPV vaccines has been a breakthrough in preventing HPV-related diseases (5–7). These vaccines are highly effective in preventing HPV infections and the subsequent development of cancer. The WHO recommends that all girls and young women aged 9–14 years receive two doses of the HPV vaccine as part of routine immunization programs (8). It is also recommended for girls and women aged 13 through 26 who have not yet been vaccinated or completed the vaccine series (9). Older people could be vaccinated, although this strategy is thought to be less effective given that this age group is more likely to have previously been exposed to HPV (10).

However, despite the availability and effectiveness of HPV vaccines, global vaccination rates remain suboptimal, and vaccination rates vary widely across different regions and countries (11). In some countries, vaccination rates are high, while in others they are low. Several factors contribute to vaccination hesitancy, including a lack of awareness about HPV and its consequences, concerns about vaccine safety, and cultural and religious barriers (12–15).

In Ethiopia, the HPV vaccination program is a relatively recent introduction and was introduced in December 2019 as part of the national immunization schedule for all 14-year-old girls through a school-based approach and in health centers (16). However, the uptake of the vaccine has been slow, with many girls and young women remaining hesitant to receive it (17–22). This is due to a variety of factors, including limited knowledge about HPV and its consequences (20), and concerns about vaccine safety (19), and cultural and religious beliefs that may discourage vaccination (13, 18).

Despite evidence showing barriers to HPV vaccination acceptance of youth girls in Ethiopia (17, 18, 20, 22, 23), most of the studies focused on knowledge and attitude-related factors and failed to comprehensively address other important potential barriers that influence vaccination acceptance in youth girls such as safety concerns and misconceptions about the HPV vaccine, parental and peer-related concerns, and cultural and religious factors. This study aims to assess hesitancy and associated factors and potential perceived barriers to HPV vaccination. By identifying the barriers to HPV vaccination in Ethiopia, policymakers and healthcare providers can develop targeted interventions to address these challenges and increase HPV vaccination acceptance.

## Methods

### Study design, setting, and samples

An institutional-based cross-sectional study was employed among female medical and health science students at the University of Gondar (UoG) from 17 July 2022 to 30 August 2022. The UoG has five campuses, such as Atse Tewodros, Atse Fasil, the College of Medicine and Health Sciences (CMHS), Maraki, and Teda campuses. At present, with nine academic offices, it offers about 87 undergraduate and 167 postgraduate programs in regular, distance, extension, and summer programs.

All selected regular undergraduate female students in the College of Medicine and Health Sciences were used as a study population. Participants who were interested in completing the self-administered questionnaire were included in the study and those unable to take part in data collection because of illness or other problems were excluded.

### Sample size determination and sampling technique

The sample size was calculated using the single population proportion formula: n = (Z *α*/2)^2^*P *(1-P)/D^2^. This is where 1.96 was used for Z α/2, 50% for P, and 5% for D because there is no previous study in the study area. Where: D is the margin of error; P is the proportion of an outcome variable; and Z α/2 is the 95% confidence interval.

After considering a 10% non-response rate, the final sample size was 424. Study participants were included in the study using a simple random sampling technique.

### Study variables

#### Dependent variable

HPV vaccine hesitancy is the main outcome variable in this study, which is categorized as hesitant and not hesitant.

#### Independent variables

Independent variables include sociodemographic variables (age, religion, residence, marital status, sex experience, department of current study, year of study, academic performance, economic status, mother’s education status, father’s education status, family income monthly, family can afford the vaccine, family history of the HPV vaccine), knowledge, and attitudes of HPV infection and vaccinations.

### Data collection instruments and procedure

Data were collected using a self-administered structured questionnaire adopted after reviewing previous literature on the topic (18, 22–25). Two data collectors with previous experience in data collection were recruited. The questionnaire consisted of sociodemographic characteristics and questions that were used to assess participants’ hesitancy of HPV vaccinations; knowledge; attitudes toward cervical cancer, HPV infection, and HPV vaccination, and potential perceived barriers to HPV vaccination (Supplementary file 1).

#### Hesitancy to receive the HPV vaccine

Vaccine hesitancy is a delay or refusal to accept vaccines, even when they are readily available. The hesitancy of the HPV vaccination was determined directly from the respondents’ responses, which asked for their willingness to accept the HPV vaccine (18). This item consisted of three questions related to previous vaccination status, current acceptance behavior, and their recommendation for families to receive HPV vaccines with a response of yes and no. The item’s internal reliability was assessed using a Kuder–Richardson Formula 20 (KR-20) and found to be in an acceptable range of 0.74.

#### Knowledge of HPV and HPV vaccine

The knowledge of participants was assessed using a total of 15 questions of knowledge-related questions, specifically related to cervical cancer, HPV infection, and HPV vaccine. Participants are expected to respond Yes (score = 1) and No (score = 0) with a maximum possible score of 15. The were five questions for assessing knowledge of cervical cancer, five questions for knowledge of HPV infection, and five questions for the knowledge of HPV vaccine. The total score was calculated and those with mean and below mean scores were considered as having poor overall knowledge, while those with the above mean score were displayed as having good overall knowledge (23, 24). A KR-20 score of the items was found to be 0.71 and showed good internal reliability of the constructed items.

#### Attitude of HPV and HPV vaccine

Attitude towards HPV vaccination was measured using seven items that assessed attitudes of participants related to cervical cancer, HPV infection, and HPV vaccines on a five-point Likert scale: one for strongly disagreeing to five for strongly agree. Attitude scores for each participant then were calculated and attitudes towards HPV ranged from 1 to 7. Participants’ attitude was categorized as follows: negative if they obtained a score of less than the mean score and positive for those who obtained more than the mean score of the total attitude score (23). The internal reliability of the constructed items found an acceptable score of a Cronbach alpha value of 0.81.

### Data quality control

The quality of the data collection instrument was maintained. The instrument was assessed for content and face validity with expertise. It was also pretested using a 5% sample to ensure the accuracy of the responses, the clarity of the questionnaire, and applicability. The data collectors received one-day training about the purposes of the study and the ethical aspects of data collection. The collected data was checked for clarity, consistency, and cleanliness throughout the collection period.

### Data entry and statistical analysis

The data were coded and entered using EpiData version 4.6 and analyzed with SPSS version 26 statistical packages. Shapiro-Willick test (*p* > 0.05), histogram, and Q-Q plots were used to assess the normality of continuous variables. Descriptive data statistics were used to describe the demographic characteristics of the participants, and the results were presented through text, tables, figures, and graphs. Multivariable logistic regression was performed to identify associations between other sociodemographic variables and knowledge and HPV vaccination acceptance among study participants.

## Results

### Sociodemographic characteristics of study participants

A total of 423 participants were included in the current study with a 99.9% response. The mean age of the participants was 22.5 ± 6.7 years. Most students reported to have academic performance (60.8%) and economic status (60.3%). Approximately three-fourths (74%) of the participants reported a personal history of sexual intercourse. Around one-third (30%) of the participants’ families were vaccinated with the HPV vaccine (Table 1).

**Table 1:** Sociodemographic characteristics of study participants at the University of Gondar, 2022 (*N* = 423).

Variable		Frequency	Percent (%)
Age (average age = 22.5 ± 6.7 years)	<20	8	1.9
	20–24	343	81.1
	25–30	72	17
Religion	Orthodox	290	68.6
	Muslim	77	18.2
	Protestant	40	9.5
	Catholics	16	3.8
Residence	Urban	345	81.6
	Rural	78	18.4
Marital status	Marriage	71	16.8
	Single	352	83.2
Sex experience	Yes	110	26
	No	313	74
Department	Medicine	175	41.4
	Physiotherapy	14	3.3
	Anesthesia	11	2.6
	Pharmacy	35	8.3
	Public health	27	6.4
	Environmental and occupational health	26	6.1
	Optometry	20	4.7
	Medical laboratory	9	2.1
	Midwifery	38	9
	Nursing	58	13.7
	Health informatics	10	2.4
Year of study	2nd year	76	18
	3rd year	137	32.4
	4th year	130	30.7
	5th year	58	13.7
	6th year	22	5.2
Academic performance	Excellent	48	11.3
	Good	257	60.8
	Fair	97	22.9
	Below	21	5
Economic status	Good	255	60.3
	Acceptable	101	23.9
	Poor	67	15.8
Mother’s education status	No education	103	24.3
	Primary education	138	32.6
	Secondary education	101	23.9
	More than secondary	81	19.1
Father’s education status	No education	75	17.7
	Primary education	94	22.2
	Secondary education	118	27.9
	More than secondary	136	32.2
Family income monthly	<8,000 birr	149	35.2
	>8,000 birr	274	64.8
Family can afford the vaccine	Yes	287	67.8
	No	136	32.2
Family history of the HPV vaccine	Vaccinated	127	30
	Not vaccinated	296	70

### Participants’ level of hesitancy of the HPV vaccine

**Figure 1:**
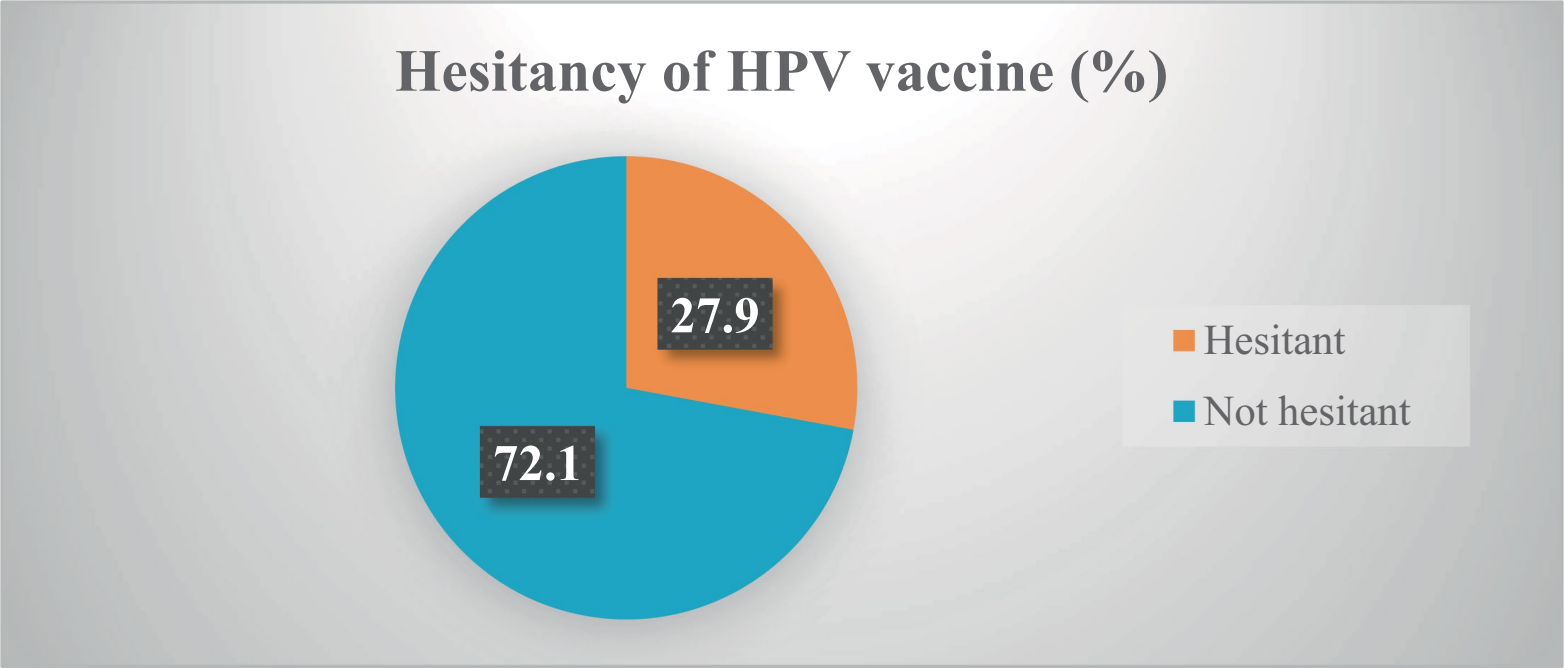
Participants’ level of hesitancy of the HPV vaccine.

Of the 423 participants, only 149 (35.2, 95% CI: 27.2–44.1) received the HPV vaccine before the survey. Currently, more than one-fourth (27.9, 95% Cl: 21.4–33.8) of participants were hesitant to receive the HPV vaccine. About two-fifths (38.5, 95% CI: 31.4–44.9) of respondents were not confidently informing and recommending their family members and relatives to get the vaccines (Table 2) and (Figure 1).

**Table 2:** Participants’ level of acceptance of the HPV vaccine (*N* = 423).

Variable		Frequency	Percent (%)
Received HPV vaccine before this survey.	Yes	149	35.2
	No	274	64.8
Are you currently willing to receive the HPV vaccine if you get the chance of having an HPV vaccine for free?	Yes	305	72.1
	No	118	27.9
Encourage your family members, and relatives, to get a vaccine	Yes	260	61.5
	No	173	38.5

### Associated factors of HPV vaccination acceptance

Multivariable logistic regression analysis showed that vaccine acceptability was associated with the participants’ monthly income, attitude, and knowledge of the HPV vaccine. HPV vaccine acceptance was significantly associated with higher monthly income (AOR = 1.52, 95% CI: 1.08–6.34), good knowledge of participants about cervical cancer, HPV infection, and HPV vaccine (AOR = 2.12, 95% CI: 1.12–4.87), and a positive attitude towards HPV vaccine (AOR = 3.03, 95% CI: 1.63–9.56) (Table 3).

**Table 3:** Factors associated with acceptance of the HPV vaccine.

Variable		HPV vaccine acceptance		95% CI		P-value
		Yes	No	COR	AOR	
Residency	Urban	249	96	1.02 (0.08–3.56)	0.78 (0.05–4.16)	0.357
	Rural	56	22	Reference	-	
Family’s monthly income	≥ 8,000	208	66	1.70 (014–4.59)	1.52 (1.08–6.34)	0.04*
	< 8,000	97	52	Reference	1	
Family history of the HPV vaccine	Vaccinated	92	35	1.02 (0.05–4.06)	0.67 (0.07–7.45)	0.381
	Not vaccinated	213	83	Reference	-	
Knowledge of cervical cancer, HPV infection, and vaccine	Good	182	70	1.55 (1.52–4.56)	2.12 (1.56–4.87)	< 0.001*
	Poor	123	48	Reference	1	
Attitude on HPV infections and vaccine	Positive	166	64	1.55 (1.52–4.56)	3.03 (1.63–9.56)	< 0.001*
	Negative	139	54	Reference	–	

*indicates *p*-value < 0.05. Bold values: Statistically associated variables.

### Perceived barriers to the HPV vaccination

Less than two-thirds of the participants (63.1%) revealed that safety concern was the main barrier to receiving the HPV vaccine, followed by misconceptions about the HPV vaccine (42.8%) and parental concerns about the HPV vaccine (42.3%) (Table 4).

**Table 4:** Perceived potential barriers to the HPV vaccination (*N* = 423).

Variable	Categories	Percent (%)	Percent
Potential barriers to the HPV vaccination	Safety concerns	267	63.1
	Misconceptions about the HPV vaccine	181	42.8
	Parental concerns about the HPV vaccine	179	42.3
	Lack of enough information about the HPV vaccine	138	32.6
	Cultural related issues	109	25.8
	Religion-related barriers	86	20.3
	Partners/peers pressure	77	18.2
	Cost and accessibility issues	73	17.3

### Participants’ level of knowledge

**Figure 2:**
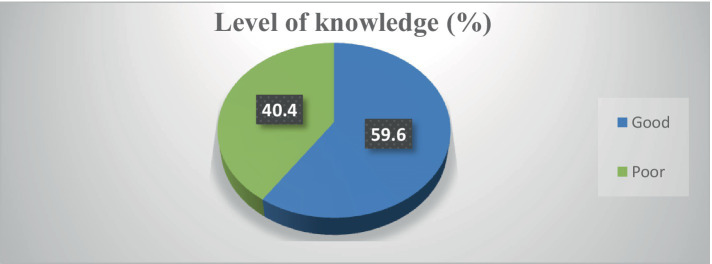
Participants’ level of knowledge about cervical cancer, HPV infection, and vaccine (*N* = 423).

Most participants heard about cervical cancer (92.9%), HPV infection (93.4%), and HPV vaccine (78%). However, most of them did not know about the risk factors of cervical cancer (87.9), risk factors of HPV infections (62.6%), and the method of prevention of HPV infection (83.9%) (Supplementary Table S1). Overall, 252 (59.6%) had good knowledge of cervical cancer, HPV infections, and vaccines (Figure 2).

### Participants’ level of attitudes

**Figure 3:**
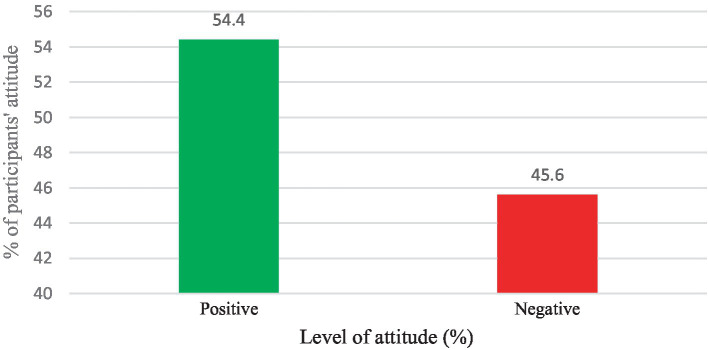
Participants’ level of attitude towards the HPV and vaccination.

In the current study, more than one-third of the participants (33.5%) agreed or strongly agreed that cervical cancer is a deadly disease. About 97.2% also strongly agreed or agreed that vaccination helps to prevent HPV infection. Moreover, about 93.2% also agreed or strongly agreed that the vaccination was beginning to minimize cervical cancer (Supplementary Table S2). Overall, most (54.4%) respondents had a positive attitude towards the HPV vaccine (Figure 3).

## Discussion

Vaccination hesitancy has continued to be a significant barrier to youths’ HPV vaccination and this study also revealed that more than one-quarter of youth girls were hesitant to receive HPV vaccines. HPV vaccination acceptance was significantly associated with the family’s monthly income, knowledge, and attitude about cervical cancer, HPV infection, and HP vaccine. This study also reported that safety concerns, misconceptions about the HPV vaccine, parental concerns about the HPV vaccine, lack of enough information about the HPV vaccine, and cultural and religious beliefs were among the top barriers to HPV vaccination of youth girls.

The findings of the current study showed that more than one-fourth of young girls are hesitant to receive the HPV vaccine currently. This figure is much lower than systematic reviews conducted on HPV vaccine uptake among adolescents in Ethiopia (17, 19, 26). A study conducted in South Africa (27), China (28), and other primary studies in Ethiopia (18, 20–22) also reported higher hesitancy levels than this study. This discrepancy could be due to differences in sociodemographic characteristics and health literacy. The study participants in the current study were health science students who were close to medical information, and they might be more aware of the importance of the HPV vaccine. The participants in China’s study were also medical students, however, the methods of hesitancy scoring are different in that this study directly asked respondents about their willingness to vaccine acceptance, while the previous study calculated from composite scores of three components such as complacency, convenience, and confidence (28). However, the current finding is much higher as compared to other studies reported in China which ranged from 10.3 to 15.5% (29, 30). This could be because of differences in socioeconomic status and access to healthcare and information, which can have an impact or contribute to vaccine acceptance or hesitancy. This finding suggests that vaccine hesitancy continues to be a public challenge and seeking tailored interventions considering contributing factors and potential barriers to vaccination uptake.

Consistent with earlier studies, parental monthly income was associated with acceptance of the HPV vaccine. Those participants with lower monthly incomes were less willing to vaccinate against the HPV vaccine compared to those with higher incomes (31). This justifies that the HPV vaccine can be expensive, especially when multiple doses are required. Lower-income families may struggle to afford this out-of-pocket expense, even if insurance partially covers it. However, in an Ethiopian context, this vaccination service is among the exempted healthcare services aiming to promote health service access. Therefore, public awareness creation could be important regarding the cost of the services.

This study also revealed that the knowledge and attitude of youth girls about cervical cancer, HPV infection, and HPV vaccines were determinant factors for HPV vaccine acceptance. These findings are consistent with other studies conducted in Ethiopia (17, 19, 20, 32) and Jordan (33). Participants with a positive attitude towards the HPV vaccine increased their chances of vaccine uptake by three times. This is consistent with a study conducted in Ethiopia (31) and Thailand (34). This might be because individuals who have a positive attitude towards the vaccine may have a higher intention of receiving the vaccine. Participants who had good knowledge of the HPV vaccine increased their vaccination uptake by about two-fold. This is a consistent study conducted in Nigeria (35) and Uganda (36). The findings highlight the importance of public awareness creation to enhance HPV vaccine acceptance in hesitant youth girls.

In the current study, safety concerns, misconceptions, and lack of enough information about the HPV vaccine were reported as the main barriers to HPV vaccination. This finding is consistent with earlier evidence (14, 15, 37, 38). This could suggest that healthcare providers should address specific concerns about vaccine safety and provide reassurance based on scientific evidence. Moreover, awareness creation campaigns may help dispel myths and provide evidence-based information about HPV, its risks, and the benefits of vaccination. For instance, awareness creation campaigns focusing on cancer prevention and HPV vaccine knowledge have resulted in reduced misinformation and increased vaccination uptake (39, 40). In addition, vaccination promotion campaigns implemented in schools and integrating HPV vaccination as school routine vaccination increased vaccination acceptance (39, 41). Furthermore, effective eHealth communication messages and delivery methods could have a positive impact on increasing the uptake of HPV vaccinations in adolescents (42).

Parental concerns about the HPV vaccine were among the major barriers to receiving HPV vaccines among youth girls. Pooled evidence meta-analysis study also revealed that parental willingness to vaccinate their children was lower (19, 43–45). This finding highlights the importance of engaging parents and caregivers in the decision-making process to build trust and address their concerns. Clear and effective communication with parents or caregivers can play a crucial role in supporting them as they decide about vaccinating their children (46).

Cultural and religious beliefs were also among the reported potential barriers to HPV vaccination of youth girls. This is in line with previous studies (13, 47, 48). The finding underscores the need for vaccination campaign interventions to address cultural and religious beliefs, emphasizing the safety and effectiveness of the vaccines (49). Furthermore, a detailed exploration of the impacts of cultural and religious beliefs could be conducted using qualitative data.

## Strengths and limitations of the study

This study presents a comprehensive finding regarding current vaccination status and their willingness to receive HPV vaccination among university students using a relatively larger sample. However, it has some limitations. Firstly, the study was conducted among only health science students, which is difficult to conclude for all young girls in Ethiopia. Secondly, this study focused only on HPV cancers. Additionally, despite HPV being recommended for males globally the current study accounts only for young girls. Furthermore, the nature of data collection, a self-administered survey, causes a social disability bias, and the outcome might be overestimated. Finally, the causal relationship might be difficult to estimate using a cross-sectional study. As a result, the authors recommend a prospective follow-up study that incorporates qualitative exploration to understand potential barriers to the vaccination of youths.

### Implication of the study

This study has implication highlights for important parties in this area such as healthcare providers, policymakers, and researchers.

#### Healthcare providers

Healthcare providers should be equipped with accurate and up-to-date information about HPV, its risks, and the benefits of vaccination. They should be able to effectively communicate this information to young girls and their parents, addressing common concerns and misconceptions. Providers should offer personalized counseling to address individual concerns and provide tailored information based on the patient’s needs and cultural background. Establishing a trusting relationship with patients is essential. Providers should be empathetic, respectful, and patient in addressing concerns and building trust. Healthcare providers can also play a crucial role in advocating for HPV vaccination policies and programs, both at the local and national levels.

#### Policymakers

Policymakers should allocate sufficient funding for HPV vaccination programs, including education campaigns, vaccine procurement, and healthcare provider training. While mandating vaccination may be controversial, it can be a powerful tool for increasing coverage. Policymakers should carefully consider the potential benefits and drawbacks of mandatory vaccination. Implementing school-based vaccination programs can make it easier for young girls to receive the vaccine and improve access in underserved communities. Policymakers should also ensure that HPV vaccination programs are equitable and accessible to all young girls, regardless of their socioeconomic status, race, or ethnicity.

#### Researchers

Further research is needed to better understand the factors contributing to HPV vaccination hesitancy, particularly in diverse populations. This research can inform the development of more effective interventions. Longitudinal studies can track changes in vaccine hesitancy over time and evaluate the effectiveness of different interventions. Qualitative research can provide valuable insights into the perspectives and experiences of young girls, parent concerns, and their decisions regarding HPV vaccination and the potential impacts of cultural and religious beliefs. Researchers should also ensure that their studies are culturally competent and consider the diverse cultural and religious beliefs of different populations. Since HPV also causes other cancers and genital warts, future studies should consider including questions about additional HPV-related diseases. Furthermore, as HPV vaccination is administered to males globally, it would be valuable to include males in such studies to better inform and target future vaccination efforts.

## Conclusion

The current study revealed that more than one-quarter of young girls are hesitant to receive the HPV vaccine, indicating that it remains a persistent challenge in today’s context. Interventional programs aiming to increase HPV vaccination in youth girls should focus on promoting awareness among youths and the public about the impact of HPV infection and the safety and importance of the HPV vaccine. Tailored interventions addressing parental concerns, and religious and cultural beliefs are also crucial to enhancing HPV vaccination in youth girls.

## Data Availability

The data supporting the conclusions of this article will be made available by the correspondence author upon reasonable request, without undue reservation.
