# Supplementary material for: Human papillomavirus vaccination hesitancy among young girls in Ethiopia: factors and barriers to uptake
Source: Front Public Health. 2025 Jan 23;13:1507832. doi: 10.3389/fpubh.2025.1507832 (PMC11798796; doi:10.3389/fpubh.2025.1507832)
Supplement: Supplementary file 1 [file Data_Sheet_1.PDF]

Data collection tool

Section I: Sociodemographic variables

Variables	Categories
Age years	1. <20
	2. 20-24
	3. 25-30
	4. > 30
Religion	1. Orthodox
	2. Muslim
	3. Protestant
	4. Catholics
Residence	1. Urban
	2. Rural
Marital status	3. Marriage
	4. Single
Sex experience	1. Yes
	2. No
Department	1. Medicine
	2. Physiotherapy
	3. Anesthesia
	4. Pharmacy
	5. Public health
	6. Environmental and occupational health
	7. Optometry
	8. Medical Laboratory
	9. Midwifery
	10. Nursing
	11. Health informatics
Year of Study	1. 2nd year
	2. 3rd year
	3. 4th year
	4. 5th year
	5. 6th year
Academic performance	1. Excellent
	2. Good
	3. Fair
	4. Below
Economic status	1. Good
	2. Acceptable
	3. Poor
Mother's education status	1. No education
	2. Primary education
	3. Secondary education
	4. More than secondary
Father's education status	1. No education
	2. Primary education
	3. Secondary education
	4. More than secondary
Family income monthly	1. <8000 birr

	2. >8000 birr
Family can afford the vaccine	1. Yes
	2. No
Family history of the HPV vaccine	1. Vaccinated
	2. Not vaccinated

Section II: HBV vaccination hesitancy

**Questions**	**Responses**
Did you receive the HPV vaccine before this survey	1. Yes
	2. No
Are you currently willing to receive the HPV vaccine if you get the chance of having an HPV vaccine for free?	1. Yes
	2. No
Encourage your family members, and relatives, to get a vaccine	1. Yes
	2. No

Section III: Knowledge of cervical cancer, HPV, and HPV vaccine

Questions	Responses
**Knowledge of cervical cancer 12**	
Have you ever heard of cervical cancer?	Yes
	No
Risk factors for cervical cancer	Don’t know
	Know
Signs and symptoms of cervical cancer	Know
	Don’t know
Sexual mode of transmission	Know
	Don’t know
Vertical mode of transmission	Know
	Don’t know
**Knowledge of HPV infection 13**	
Have you ever heard of HPV infection	Yes
	No
Who can contract HPV infection?	Know
	Don’t know
Diseases caused by HPV infection	Know
	Don’t know
Risk factors for HPV infection	Know
	Don’t know
Method of prevention of HPV infection	Know
	Don’t
**Knowledge of HPV vaccine 7**	
Have you ever heard about the HPV vaccine before?	Yes
	No
Who should get the HPV vaccination?	Know
	Don’t know
Did you know that the HPV vaccines can prevent cervical cancer and warts?	Know
	Don’t know
Recommended doses of HPV vaccine	Know
	Don’t know
The ideal time HPV Vaccine is best recommended	Know
	Don’t know

Section IV: Attitudes to cervical cancer, HPV infection, and HPV vaccines

Questions	Responses
Cervical cancer is a deadly disease	1. Strongly disagree<br>2. Disagree<br>3. Neutral<br>4. Agree<br>5. Strongly agree
Believe vaccination helps to prevent HPV infection	1. Strongly disagree<br>2. Disagree<br>3. Neutral<br>4. Agree<br>5. Strongly agree
The vaccination was beginning to minimize cervical cancer	1. Strongly disagree<br>2. Disagree<br>3. Neutral<br>4. Agree<br>5. Strongly agree
Parental concerns about having the vaccine	1. Strongly disagree<br>2. Disagree<br>3. Neutral<br>4. Agree<br>5. Strongly agree
HPV vaccine saves lives and improves health	1. Strongly disagree<br>2. Disagree<br>3. Neutral<br>4. Agree<br>5. Strongly agree
Whether you recommend the vaccine to others or not	1. Strongly disagree<br>2. Disagree<br>3. Neutral<br>4. Agree<br>5. Strongly agree
Having the HPV Vaccine may become sexually promiscuous	1. Strongly disagree<br>2. Disagree<br>3. Neutral<br>4. Agree<br>5. Strongly agree

Section V: potential perceived barriers to the HPV vaccination

**Potential barriers to the HPV vaccination**	Yes	No
Parental concerns about the HPV vaccine		
Lack of enough information about the HPV vaccine		
Safety Concerns		
Misconceptions about the HPV vaccine		
Partners/peers pressure		
Cost and accessibility issues		
Cultural related issues		
Religion-related barriers		
Others. please mention if		
