# Supplementary material for: Human papillomavirus vaccination hesitancy among young girls in Ethiopia: factors and barriers to uptake
Source: Front Public Health. 2025 Jan 23;13:1507832. doi: 10.3389/fpubh.2025.1507832 (PMC11798796; doi:10.3389/fpubh.2025.1507832)
Supplement: Supplementary file 2 [file Table_1.DOCX]

| **Variables** | | | **Frequency** | **Percent (%)** |
| --- | --- | --- | --- | --- |
| **Knowledge of cervical cancer** | | |  |  |
| Have you ever heard of cervical cancer? | | Yes | 393 | 92.9 |
|  |  | No | 30 | 7.1 |
| Risk factors for cervical cancer | | Don’t know | 372 | 87.9 |
|  |  | Know | 51 | 12.1 |
| Signs and symptoms of cervical cancer | | Know | 340 | 80.4 |
|  |  | Don’t know | 83 | 19.6 |
| Mode of transmission | Sexual transmission | Know | 372 | 87.9 |
|  |  | Don’t know | 51 | 12.1 |
|  | Vertical transmission | Know | 340 | 80.4 |
|  |  | Don’t know | 83 | 19.6 |
| **Knowledge of HPV infection** | |  |  |  |
| Have you ever heard of HPV infection | | Yes | 395 | 93.4 |
|  |  | No | 28 | 6.6 |
| Who can contract HPV infection? | | Know | 364 | 86.1 |
|  |  | Don’t know | 59 | 13.9 |
| Diseases caused by HPV infection. | | Know | 347 | 82 |
|  |  | Don’t know | 76 | 18 |
| Risk factors for HPV infection | | Know | 158 | 37.4 |
|  |  | Don’t know | 265 | 62.6 |
| Method of prevention of HPV infection | | Know | 68 | 16.1 |
|  |  | Don’t | 355 | 83.9 |
| **Knowledge of HPV vaccine** | |  |  |  |
| Have you ever heard about the HPV vaccine before? | | Yes | 330 | 78 |
|  |  | No | 93 | 22 |
| Who should get the HPV vaccination? | | Know | 233 | 55.1 |
|  |  | Don’t know | 190 | 44.9 |
| Did you know that the HPV vaccines can prevent cervical cancer and warts? | | Know | 245 | 57.9 |
|  |  | Don’t know | 178 | 42.1 |
| Recommended doses of HPV vaccine | | Know | 15 | 3.5 |
|  |  | Don’t know | 408 | 96.5 |
| The ideal time HPV Vaccine is best recommended | | Know | 101 | 23.9 |
|  |  | Don’t know | 322 | 76.1 |
