# Supplementary material for: Human papillomavirus vaccination hesitancy among young girls in Ethiopia: factors and barriers to uptake
Source: Front Public Health. 2025 Jan 23;13:1507832. doi: 10.3389/fpubh.2025.1507832 (PMC11798796; doi:10.3389/fpubh.2025.1507832)
Supplement: Supplementary file 3 [file Table_2.DOCX]

| **Response question** | **Percent responses (n, %)** | | | | |
| --- | --- | --- | --- | --- | --- |
|  | Strongly agree | Agree | Neutral | Disagree | Strongly disagree |
| Cervical cancer is a deadly disease | 141(33.3%) | 1 (0.2%) | 266 (62.9%) | 15 (3.5%) | - |
| Believe vaccination helps to prevent HPV infection | 227 (53.7%) | 184 (43.5%) | 4(0.9%) | 3 (0.7%) | 5 (1.2%) |
| The vaccination was beginning to minimize cervical cancer | 203 (48.0%) | 191 (45.2%) | 21(5.0%) | 3 (0.7%) | 5 (1.2%) |
| Parental concerns about having the vaccine | 66 (15.6%) | 74 (17.5%) | 189 (44.7%) | 61(14.4%) | 33 (7.8%) |
| HPV vaccine saves lives and improves health | 46 (10.9%) | 59(13.9%0 | 158 (37.4%) | 34 (8.0%) | 126 (29.8%) |
| Whether you recommend the vaccine to others or not | 126 (29.8%) | 123 (29.1%) | 117(27.7%) | 45 (10.6%) | 12 (2.8%) |
| Having the HPV Vaccine may become sexually promiscuous | 17 (4.0%) | 39 (9.2%) | 141 (33.3%) | 122 (28.8%) | 104 (24.6%) |
